# Clinical efficacy and safety of topiroxostat in Japanese hyperuricemic patients with or without gout: a randomized, double-blinded, controlled phase 2b study

**DOI:** 10.1007/s10067-016-3474-8

**Published:** 2016-11-10

**Authors:** Tatsuo Hosoya, Tomomitsu Sasaki, Tetsuo Ohashi

**Affiliations:** 1 Department of Pathophysiology and Therapy in Chronic Kidney Disease, Jikei University School of Medicine, Tokyo, Japan; 2 Development Department, Medical R&D Division, Fuji Yakuhin Co., Ltd., 4-383, Sakuragi-cho, Omiya-ku, Saitama-shi, Saitama 330-9508 Japan

**Keywords:** Gout, Hyperuricemia, Late phase 2 clinical study, Topiroxostat, Xanthine oxidoreductase inhibitor

## Abstract

Topiroxostat, a selective xanthine oxidoreductase inhibitor, is used in Japan for the treatment of hyperuricemic patients with or without gout. In terms of the effectiveness of topiroxostat in lowering serum urate levels, the dose–response relationship has been evaluated; however, it remains to be verified. A randomized, multi-center, double-blinded study of topiroxostat was performed for Japanese hyperuricemic patients with or without gout. During the 16-week study, 157 Japanese hyperuricemic patients with or without gout were randomly assigned to receive a placebo, topiroxostat at 120 or 160 mg/day, or allopurinol at 200 mg/day. The primary endpoint of this study was to determine the lowering rate of serum uric acid levels compared to those of baseline at the end of administration. A dose–response relationship (regarding decreases in the serum urate levels) was confirmed for the placebo and topiroxostat at 120 and at 160 mg/day. Moreover, at the end of administration, the lowering rate of serum urate levels was determined to be −44.8% in the topiroxostat 160-mg/day group. No significant difference in the incidence of adverse events was observed among all groups, including the allopurinol group. The serum urate-lowering effect of topiroxostat was found to have a dose–response relationship in Japanese hyperuricemic patients with or without gout.

**Electronic supplementary material:**

The online version of this article (doi:10.1007/s10067-016-3474-8) contains supplementary material, which is available to authorized users.

## Introduction

Hyperuricemia [defined by a serum urate concentration ≥416.4 μmol/L (or ≥7.0 mg/dL) in Japan] is a causative factor of urate deposition disease (e.g., urolithiasis and gouty arthritis). From the perspective of gout prevention, the main goal of hyperuricemia treatment is to lower serum urate levels and to maintain the levels at ≤356.9 μmol/L (or ≤6.0 mg/dL) [1–3]. Meanwhile, in recent years, hyperuricemia has been found to cause renal impairment and to be associated with the onset and progression of chronic kidney disease (CKD) [4–6]. Several interventional studies have suggested that serum urate-lowering agents, such as allopurinol, effectively maintain renal function in CKD [7, 8]. For asymptomatic hyperuricemia, in cases with underlying diseases, such as hypertension, ischemic heart disease, and kidney disease, serum urate levels of ≥475.8 μmol/L (or 8.0 mg/dL) have been established as the criteria for the introduction of pharmacotherapy in Japan [1].

Allopurinol [a hypoxanthine-analog xanthine oxidoreductase (XOR) inhibitor] is a serum urate-lowering agent widely used throughout the world. However, side effects, such as allergies and liver impairment, and even severe rash associated with myelosuppression and necrosis have been reported, albeit rarely. Furthermore, since allopurinol and its active metabolite oxipurinol are mainly excreted via the kidneys, there is a concern among patients with impaired renal function that they may cause an elevation in serum oxipurinol levels, thereby causing a higher incidence of side effects. Therefore, the dosage should be regulated [9–11].

Topiroxostat (formerly known as FYX-051) is a non-purine selective XOR inhibitor [12–14]. Unlike chemical structure-based XOR inhibitors (e.g., febuxostat), topiroxostat is a hybrid-type inhibitor that not only has a chemical structure-based XOR inhibitory action but also binds covalently to molybdenum in the active center during the enzyme-induced hydroxylation process [12, 13]. In addition, the pharmacokinetics of unchanged topiroxostat or its metabolites is unaffected by mild-to-moderate renal impairment, and in patients with concurrent moderate renal impairment and hyperuricemia, it has been reported to lower the serum urate levels and urinary albumin levels [15].

The present study was performed to evaluate and verify the serum urate-lowering effects, the dose–response relationship in terms of serum urate reduction, and the safety of topiroxostat in hyperuricemic patients with or without gout.

## Materials and methods

### Study design

The present study was performed to verify the dose–response relationship and optimal dose of topiroxostat for lowering serum urate levels. Before the start of the study, the institutional review board of each participating institution provided approval after reviewing the study content. Furthermore, this study was conducted in accordance with the Declaration of Helsinki, Good Clinical Practice guidelines, and other relevant regulations. Written informed consent was obtained from all study participants prior to the start of the pre-observation period, after providing a thorough explanation of the study content.

This study is a phase 2, multi-center, randomized, placebo-controlled, double-blind verification study. Fourteen clinical institutions of Japan participated in the study.

**Fig. 1:**
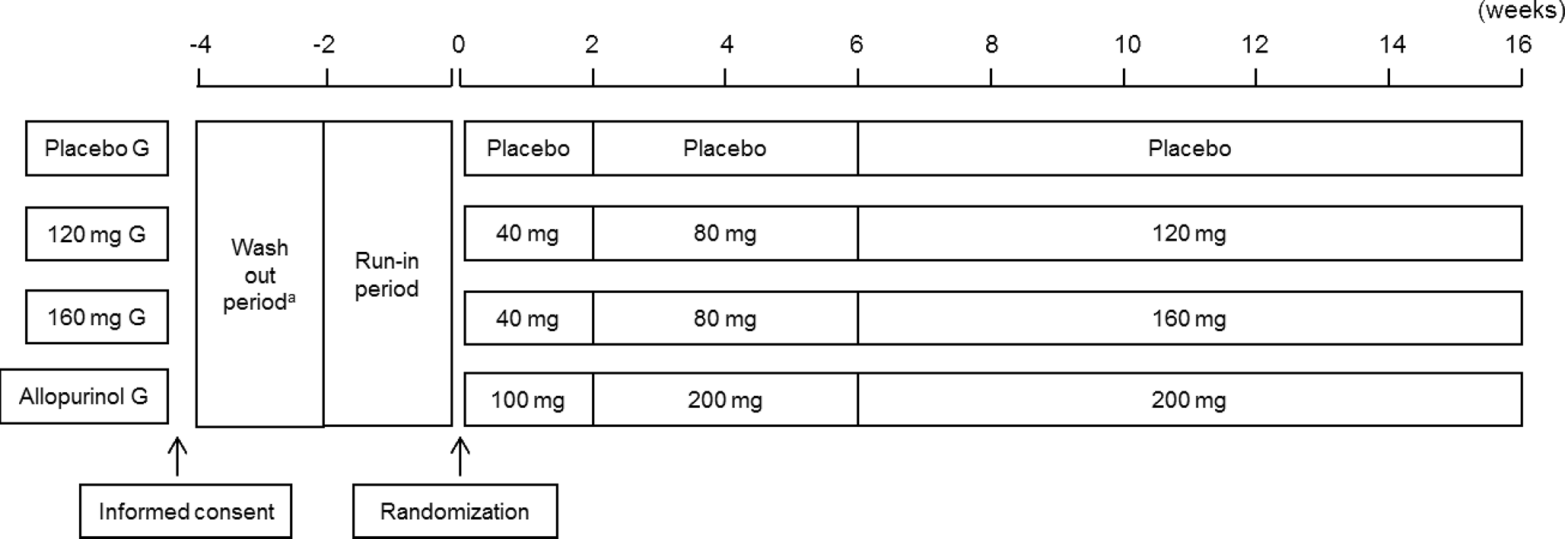
Study schema. ^a^In this case, patients had been prescribed urate-lowering agents or agents affecting the serum urate level

Patients were randomly assigned to either placebo, topiroxostat 120/160-mg, or allopurinol 200-mg daily group (ratio 1:1:1:1). The allopurinol arm was also set up as a reference to examine the effectiveness, safety, and incidence of gouty arthritis. To minimize the risk of gouty arthritis developing as a result of a sudden reduction in serum urate levels [16], the dose titration method was used. The dosage scheme is shown in Fig. 1. This study consisted of a run-in period (1 to 4 weeks) and a treatment period (initial phase I, 2 weeks; initial phase II, 4 weeks; maintenance phase, 10 weeks).

In the event that patients had been receiving therapy with drugs thought to affect serum urate levels prior to enrollment, the patients were enrolled after a washout period of at least 2 weeks. To reduce the difference between groups according to patient attributes, dynamic allocation was used. Factors taken into consideration for dynamic allocation included “medical institution participating in the study,” “serum urate level (value measured on the closest day to the start of treatment),” and “the presence or absence of a history of gouty arthritis.”

For the classification of hyperuricemia, based on urate measurement in the 60-min urine collection during the run-in period, we classified patients into the following four types: (1) patients with urinary excretion of urate (EUA; mg/kg/h) >0.51 and urate clearance (CUA; mL/min) ≥7.2 were defined as “urate-overproduction type,” (2) patients with EUA <0.48 or CUA <7.2 were defined as “urate-underexcretion type,” (3) patients with EUA >0.51 and CUA <7.2 were defined as “combined type,” and (4) patients with 0.48 ≤ EUA ≤0.51 and CUA ≥7.2 were defined as “normal type”.

### Inclusion and exclusion criteria

Patients were included in this study if they met the following inclusion criteria: Japanese patients aged 20 to 64 years who were able to provide consent in writing, with serum urate level in the run-in period of ≥416.4 μmol/L in patients with tophi or a history of gout attacks or ≥535.4 μmol/L in patients with hyperuricemia (however, ≥475.8 μmol/L in patients who were receiving treatment for or had a diagnosis of urolithiasis, hypertension, hyperlipidemia, or diabetes).

Patients were excluded from the study if they met the following exclusion criteria: the onset of gouty arthritis within 2 weeks prior to the start of the study drug administration; primary or secondary hyperuricemia that occurs secondary to specific disorders, including Lesch–Nyhan syndrome, hematologic malignancies, or Down’s syndrome; HbA1c ≥8.0% or poorly controlled hyperglycemia; renal function impairment (eGFR ≥50 mL/min/1.73 m^2^); liver impairment (ALT ≥100 U/L and/or AST ≥100 U/L), severe hypertension (systolic blood pressure ≥180 mmHg and/or diastolic blood pressure ≥110 mmHg or poorly controlled condition with hypotensive agent); and the use of urate-lowering agents, azathioprine, 6-mercaptoprine, theophylline, the study drug other than topiroxostat, or agents thought to affect the outcomes during the period from 2 weeks prior to the start of the pre-observation period until the day of treatment commencement.

### Blinding

The individuals responsible for allocating the study drug performed all allocations and managed the allocation table (including study drug distribution). Furthermore, to maintain the double-blind condition, serum urate levels measured after administration of the study drug were concealed from the physician responsible for the treatment, the patients, and study applicants throughout the study period.

#### Endpoints

The primary efficacy endpoint was the serum urate reduction rate from baseline values upon the final visit. The secondary endpoint was the achievement rate of serum urate level ≤356.9 μmol/L, etc., upon the final visit. In addition, for laboratory tests, samples were collected from each institution, and measurements were managed in an integrated manner.

### Safety evaluations

Any adverse events (AEs) and safety assessments conducted by clinical investigators, including vital signs, 12-lead electrocardiography, clinical laboratory tests, and clinical examinations, were recorded during the study period. AEs were classified according to system organ class and preferred term (MedDRA/J version 13.0) and were evaluated in terms of their potential relationship with the study drug and severity. Furthermore, patients who developed severe AEs discontinued the treatment. To evaluate the incidence of gouty arthritis, preventive administration of colchicine was not permitted during the pre-observation and the double-blind test period.

### Statistical analyses

In this study, the primary endpoint was the serum urate-lowering rate, which was analyzed by examining dose responsiveness of the serum urate-lowering rates using the Jonckheere–Terpstra test. To achieve this, the number of patients who gave the Jonckheere–Terpstra test detection power of 80% or above was used as the target number of patients to calculate the rates. In other words, on the basis of the results of the two phase 2 (2a) trials, in this study, the serum urate-lowering rates of each topiroxostat treatment group (placebo group, 120-mg group, and 160-mg group) were expected to be 0, 39, and 47%, respectively, with an assumed standard deviation of 9%, and when calculating the number of patients in the dose–response study according to a simulation with a 5% level of significance and detection power of 80%, there were three patients per group. Taking into consideration the number of patients who may be excluded from the analysis and the number of patients in whom safety could be evaluated, we set the target number of patients as 40 patients per group.

Analysis sets for efficacy included the per protocol set (PPS) and full analysis set (FAS), the latter consisting of all the randomized patients who received at least one dose of the study drug and underwent serum urate measurement during at least one visit, and primary analyses were performed for the FAS. To impute the missing value of serum urate levels, the modified last observation carried forward was used; if the serum urate data at week 16 was missed, the data at week 14 was used as the final-visit data. The approach was pre-specified before the start of the study.

The *χ* ^2^ test was used for testing the secondary efficacy endpoint, and Bonferroni correction was used to avoid the multiplicity of testing. Cochran–Armitage test was used for the evaluation of dose dependency of the secondary efficacy endpoint. In addition, we performed a pre-specified sub-group analysis of the primary efficacy endpoint by the classification of hyperuricemia.

Safety analyses were performed on the safety population, which consisted of all the patients who took at least one dose of the study drug. The incidences of AEs were summarized in the number and proportion of patients.

Statistical analyses were performed using the software Statistical Analysis System release 8.2 (SAS Institute, Cary, NC, USA). Unless otherwise stated, data are shown as mean ± standard deviation (SD). The level of significance of a two-tailed test was set at a *P* value of 5% (2.5% one way). However, for comparisons of patient attributes, a two-tailed *P* value of 15% was considered significant.

## Results

### Participant details

**Fig. 2:**
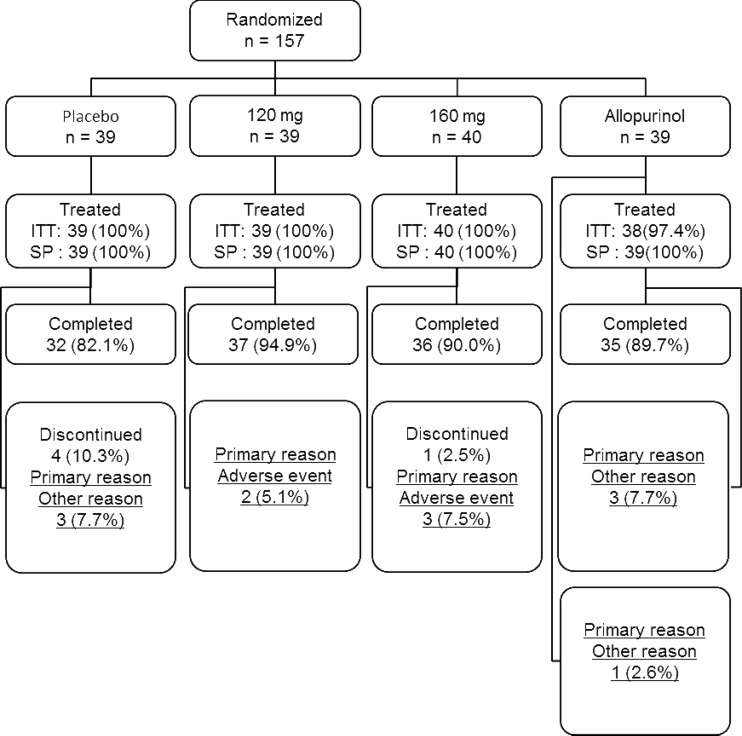
Flow diagram of the study

In this study, 223 patients signed the informed consent. A breakdown of study participants is shown in Fig. 2. The study drug was randomly allocated to 157 patients in 14 participating institutions (placebo group 39 patients, topiroxostat 120-mg group 39 patients, topiroxostat 160-mg group 40 patients, and allopurinol group 39 patients). The study treatment was completed by 34 patients in the placebo group, 39 patients in the topiroxostat 120-mg group, 39 patients in the topiroxostat 160-mg group, and 38 patients in the allopurinol group. The treatment was discontinued by five patients in the placebo group, zero patient in the topiroxostat 120-mg group, one patient in the topiroxostat 160-mg group, and one patient in the allopurinol group. Analysis of safety was performed for all 157 cases but that of efficacy was performed for 156 cases, since one case, whose emergency key had been broken before finalizing the CRF, was excluded.

### Patient attributes

Patient attributes for FAS are shown in Table 1.

**Table 1:** Baseline characteristics (full analysis set)

		Placebo (*n* = 39)	Topiroxostat 120 mg/day (*n* = 39)	Topiroxostat 160 mg/day (*n* = 40)	Allopurinol (*n* = 38)	Total	*P* value*
Sex	Male, *n*	39	38	38	38	153	*χ* ^2^ *P* = 0.308
	Female, *n*	0	1	2	0	3	
Age (year)	Mean ± SD	49.6 ± 8.1	50.7 ± 8.4	53.2 ± 7.9	52.3 ± 8.4	51.4 ± 8.2	ANOVA *F* = 1.520 *P* = 0.211
	<40	4	4	4	3	15	Kruskal–Wallis *P* = 0.014*
	≥40 < 50	17	16	7	11	51	
	≥50	18	19	29	24	90	
Height (cm)	Mean ± SD	169.43 ± 5.50	171.77 ± 6.51	169.74 ± 7.87	168.66 ± 6.24	169.90 ± 6.63	ANOVA *F* = 1.570 *P* = 0.199
	<165.0	9	2	9	13	33	Kruskal–Wallis *P* = 0.016*
	≥165.0 < 175.0	25	25	23	19	92	
	≥175.0	5	12	8	6	31	
Body weight (kg)	Mean ± SD	74.08 ± 9.12	76.28 ± 11.56	74.38 ± 12.59	74.95 ± 12.73	74.92 ± 11.51	ANOVA *F* = 0.277 *P* = 0.842
	<70.0	12	15	21	15	63	Kruskal–Wallis *P* = 0.672
	≥70.0 < 80.0	15	9	6	10	40	
	≥80.0	12	15	13	13	53	
Duration of hyperuricemia (year)	Mean ± SD	11.62 ± 8.73	10.70 ± 7.19	12.18 ± 8.89	12.46 ± 9.02	11.74 ± 8.43	ANOVA *F* = 0.325 *P* = 0.807
	<5.0	8	9	8	10	35	Kruskal–Wallis *P* = 0.991
	≥5.0 < 15.0	20	17	19	15	71	
	≥15.0	11	13	13	13	50	
Serum urate (μmol/L)	Mean ± SD	535.9 ± 69.6	539.5 ± 82.1	535.3 ± 70.8	549.6 ± 95.2	540.1 ± 79.7	ANOVA *F* = 0.252 *P* = 0.860
	≥416.4 < 475.8	7	7	8	6	28	Kruskal–Wallis *P* = 0.973
	≥475.8 < 535.3	13	14	13	13	53	
	≥535.3 < 594.8	10	9	10	9	38	
	≥594.8	9	9	9	10	37	

*P < 0.05

The 156 patients constituting the FAS included 153 males and 3 females. The patient age was 51.4 ± 8.2 years (mean ± SD), with a height of 169.90 ± 6.63 cm, weight of 74.92 ± 11.51 kg, the duration of hyperuricemia of 11.74 ± 8.43 years, and serum urate level of 540.1 ± 79.7 μmol/L.

The bias in distribution between treatment groups was observed in age (Kruskal–Wallis test *P* = 0.114) and in height (Kruskal–Wallis test *P* = 0.016).

### Efficacy

**Fig. 3:**
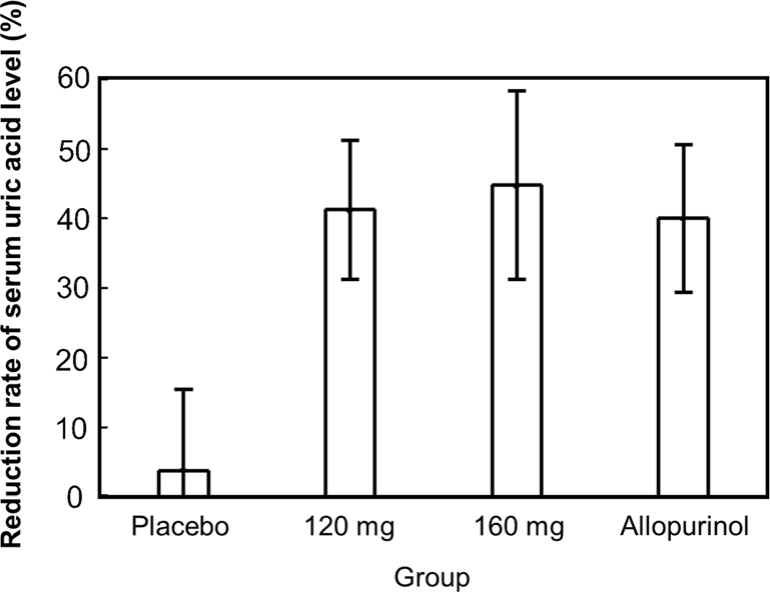
Percent reduction from baseline in serum urate level at the final visit (FAS)

Figure 3 shows the analysis results of the FAS for serum urate-lowering rate upon the final visit, which was the primary endpoint. The serum urate-lowering rate ± SD of the placebo group, topiroxostat 120-mg group, topiroxostat 160-mg group, and allopurinol group upon the final visit were 3.93 ± 11.39, 40.92 ± 9.84, 44.79 ± 13.26, and 40.18 ± 10.30%, respectively, indicating a significant urate-lowering rate in the topiroxostat 120-mg group, the topiroxostat 160-mg group, and the allopurinol group compared to that in the placebo group (Tukey’s test *P* < 0.001). Furthermore, dose responsiveness was observed in the serum urate-lowering action in the placebo group and the topiroxostat 120- and 160-mg groups (Jonckheere–Terpstra test *P* < 0.001).

**Fig. 4:**
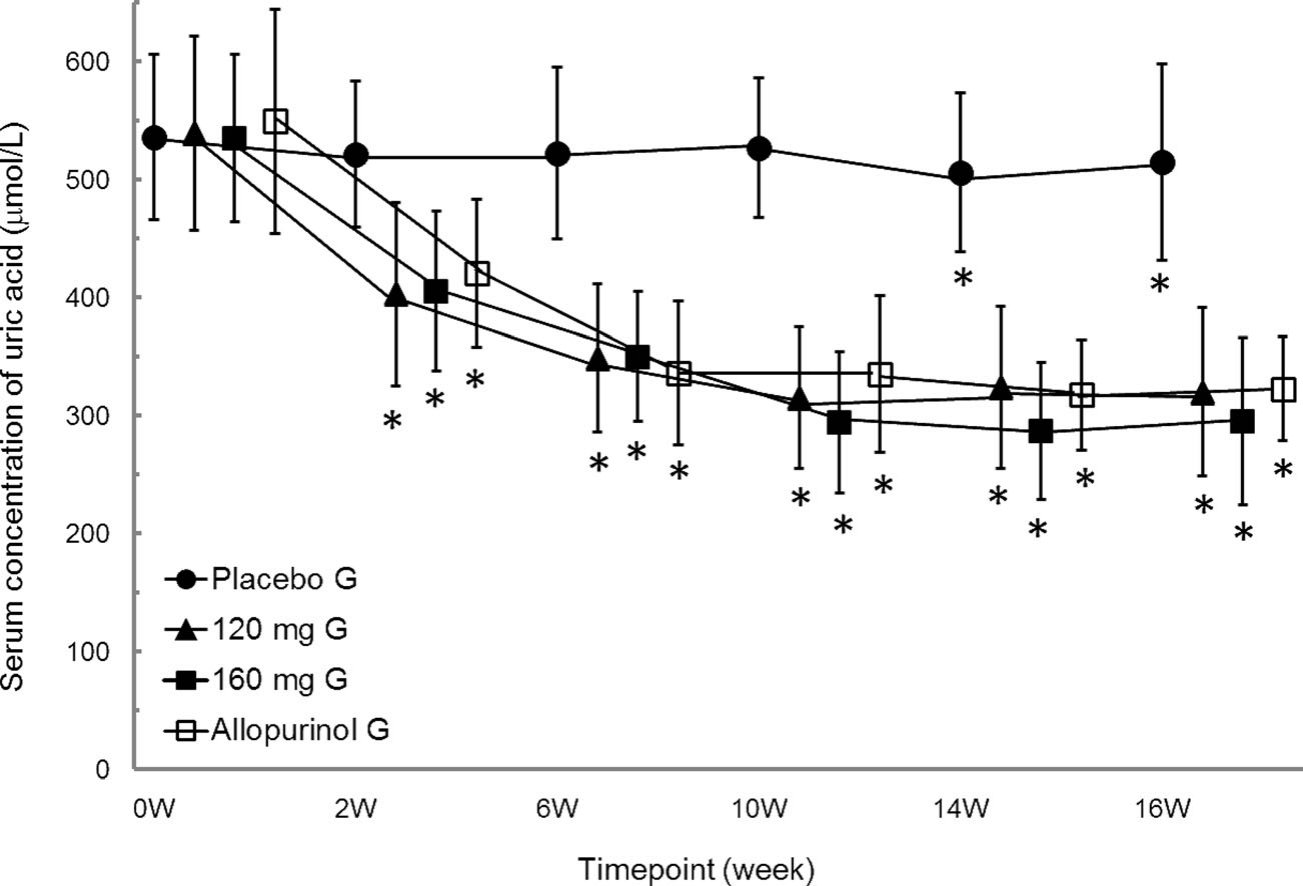
Time-course plots of serum urate level at each time point (FAS). Mean ± SD. *P < 0.05 (vs. 0 W). To convert serum urate levels from μmol/L to mg/dL, divide by 59.48

The serum urate levels for the FAS at each point in time (prior to treatment and at 2, 6, 10, 14, and 16 weeks of treatment) are shown in Fig. 4. Thus, a serum urate-lowering effect was observed with each dosage of the topiroxostat groups as well as in the allopurinol group compared to baseline values before administration.

The achievement rates of the serum urate level ≤356.9 μmol/L upon the final visit in the FAS are shown in Table S1. Upon the final visit, serum urate level of ≤356.9 μmol/L was achieved by 0/35 patients (0.0%) in the placebo group, 30/39 patients (76.9%) in the topiroxostat 120-mg group, 30/39 patients (76.9%) in the topiroxostat 160-mg group, and 32/38 patients (84.2%) in the allopurinol group. In addition, the placebo group, the topiroxostat 120-mg group, and the topiroxostat 160-mg group, in which the achievement rates of serum urate levels ≤356.9 μmol/L were obtained, exhibited a dose–response relationship (Cochran–Armitage test *P* < 0.001). Furthermore, independency between each treatment group and each achievement rate was dismissed (independency test *P* < 0.001). Although a significant difference was observed between the placebo group and the topiroxostat 120-mg group, the placebo group and the topiroxostat 160-mg group, and the placebo group and the allopurinol group (*χ* ^2^ test *P* < 0.001), no significant difference was observed between the topiroxostat 120- and 160-mg groups, the topiroxostat 120-mg group and allopurinol group, and the topiroxostat 160-mg group and the allopurinol group (*χ* ^2^ test *P* = 1.000, *P* = 0.420, and *P* = 0.420). Proportion of subjects whose serum urate level ≤297.4 μmol/L are also shown in Table S1. Because these data are not the pre-specified secondary endpoint, the data are shown as a result of post hoc analysis without statistics. Upon the final visit, serum urate level of ≤297.4 μmol/L was achieved by 0/35 patients (0.0%) in the placebo group, 15/39 patients (38.5%) in the topiroxostat 120-mg group, 23/39 patients (59.0%) in the topiroxostat 160-mg group, and 11/38 patients (28.9%) in the allopurinol group.

Amount of change in serum urate level was also shown in Table S2. For FAS, the amount of change in serum urate levels at the final visit from the level at baseline in each group (placebo group, topiroxostat 120- and 160-mg groups, and allopurinol group) (mean ± SD) were −22.4 ± 63.6, −220.8 ± 64.7, −243.0 ± 82.1, and −226.7 ± 84.9 μmol/L, respectively. A significant difference was observed between topiroxostat 120-mg group and placebo group, topiroxostat 160-mg group and placebo group, and also allopurinol group and placebo group (Tukey’s test *P* < 0.001).

### Safety

Among the AEs observed in this study, with regards to adverse effects for which a causal relationship could not be denied, the number of patients with/incidence of adverse effects including gouty arthritis was 15/39 patients (38.5%) in the placebo group, 8/39 patients (20.5%) in the topiroxostat 120-mg group, 7/40 patients (17.5%) in the topiroxostat 160-mg group, and 10/39 patients (25.6%) in the allopurinol group, with a significant difference observed between the placebo group and the topiroxostat 160-mg group (*χ* ^2^ test *P* = 0.038). Moreover, the number of patients with/incidence of adverse effects excluding gouty arthritis was 13/39 patients (33.3%) in the placebo group, 6/39 patients (15.4% in the topiroxostat 120-mg group, 6/40 patients (15.0) in the topiroxostat 160-mg group, and 7/39 patients (17.9%) in the allopurinol group, with no significant difference observed for any group (*χ* ^2^ test) (Table 2).

**Table 2:** Incidence of adverse events and/or adverse drug reactions in any treatment group

	Placebo group (*n* = 39)			Topiroxostat 120-mg group (*n* = 39)			Topiroxostat 160-mg group (*n* = 40)			Allopurinol group (*n* = 39)		
	Adverse event	Adverse drug reaction		Adverse event	Adverse drug reaction		Adverse event	Adverse drug reaction		Adverse event	Adverse drug reaction	
Number of subjects	29	15		28	8		25	7		23	10	
Incidence (%)	74.4	38.5		71.8	20.5		62.5	17.5		59.0	25.6	
*χ* ^2^ test	*P* vs. 120	*P* vs. 160	120 vs. 160	*P* vs. Allo	120 vs. Allo	160 vs. Allo	*P* vs. 120	*P* vs. 160	120 vs. 160	*P* vs. Allo	120 vs. Allo	160 vs. Allo
*P*	0.799	0.257	0.379	0.150	0.234	0.748	0.082	0.038*	0.733	0.225	0.591	0.379

*Incidence (%)* number of adverse events- and/or adverse drug reaction-observed subjects/number of subjects for safety evaluation

*P < 0.05

Severe AEs which occurred during the study included one case of death (suicide) (allopurinol group) and one case of left nephrolithiasis (placebo group). The former case was attributed to family relationship problems, and the attending physician rejected a causal relationship. For the latter case, the attending physician determined that the renal stones had already developed prior to the start of treatment with the study drug, and thus, a causal relationship was rejected. The severity of all other AEs and adverse effects were mild to moderate.

In this study, the number of patients with/incidence of gouty arthritis in each group was 3/39 patients (7.7%) in the placebo group, 2/39 patients (5.1%) in the topiroxostat 120-mg group, 2/40 patients (5.0%) in the topiroxostat 160-mg group, and 4/39 patients (10.3%) in the allopurinol group.

There were no clinically relevant changes in vital signs and 12-lead ECG.

## Discussion

In the present study, we confirmed and verified dose–response relationship for placebo and topiroxostat at 120- and 160-mg doses regarding the percent change in serum urate level from baseline to the final visit as the primary efficacy endpoint, and we also determined the percentage of patients with serum urate level ≤356.9 μmol/L at the final visit, as the secondary efficacy endpoint. In addition, as a result of prescribed sub-group analysis by the classification of hyperuricemia, topiroxostat may have serum urate-lowering efficacy beyond the classification of hyperuricemia (urate-overproduction type and urate-underexcretion type).

For safety, the majority of AEs were mild to moderate, with comparable incidences among all treatment groups. However, further long-term studies are needed to obtain more data with regards to the safety profile of topiroxostat according to the actual status of the use of therapeutic agents for hyperuricemia.

The prevention of gouty arthritis is one important goal in treatment to lower serum urate levels [1]. However, administration of XOR inhibitors rapidly reduces serum urate levels, particularly at the start of treatment [17], which sometimes promotes the onset of gouty arthritis. Therefore, we evaluated the risk of gouty arthritis onset during the topiroxostat treatment period. For this reason, the preventive use of colchicine was not permitted. Instead, the dose titration method was employed to administer the study drug. This method is recommended in Japan in the treatment of hyperuricemia from the perspective that it can avoid the onset of gouty arthritis caused by a sudden drop in serum urate levels. The number of patients with/incidence of gouty arthritis was low in this trial, with 3/39 patients (7.7%) in the placebo group, 2/39 patients (5.1%) in the topiroxostat 120-mg group, 2/40 patients (5.0%) in the topiroxostat 160-mg group, and 4/39 patients (10.3%) in the allopurinol group. Furthermore, the pharmacokinetics of topiroxostat is unaffected by mild-to-moderate renal impairment, and therefore, there is no need to adjust the administration route or dosage in patients with moderate renal impairment [15]. This is a clinically significant difference compared to allopurinol.

This study demonstrated the dose–response relationship for the placebo and topiroxostat at doses of 120 and 160 mg for serum urate-lowering rate upon the final visit, with no safety problems. Furthermore, the serum urate-lowering rate upon the final visit of allopurinol, a congener agent with comparable effects, set as a comparative reference, and the achievement rate of serum urate levels <356.9 μmol/L upon the final visit should be taken into consideration, and we believe that a controlled comparative trial of allopurinol should be performed with topiroxostat at 120 mg.

## Electronic supplementary material

Table S1 (PDF 69 kb)

Table S2 (PDF 53 kb)
