# Supplementary material for: Clinical efficacy and safety of topiroxostat in Japanese hyperuricemic patients with or without gout: a randomized, double-blinded, controlled phase 2b study
Source: Clin Rheumatol. 2016 Nov 10;36(3):649–56. doi: 10.1007/s10067-016-3474-8 (PMC5323498; doi:10.1007/s10067-016-3474-8)
Supplement: Supplementary file 1 — (PDF 69 kb) [file 10067_2016_3474_MOESM1_ESM.pdf]

**Table S1** Proportion of subjects whose serum urate level ≤356.9 μmol/L at the final visit (FAS)

| Group | n | Proportion of subjects: Number (%) of ≤ 297.4 μmol/L[#] | Proportion of subjects: Number (%) of ≤ 356.9 μmol/L | Proportion of subjects: 95% C.L. of Proportion | Statistics: $\chi^2$ test | | Statistics: Cochran-Armitage test | Statistics: Independency test |
|---|---|---|---|---|---|---|---|---|
| P | 35 | 0 (0.0) | 0 (0.0) | 0.00–8.20 | P vs. 120 | $\chi^2$ = 45.280<br>$P$ < 0.001* | Z = 6.500<br>$P$ < 0.001 * | $\chi^2$ = 71.613<br>$P$ < 0.001* |
| | | | | | P vs. 160 | $\chi^2$ = 45.280<br>$P$ < 0.001* | | |
| 120 | 39 | 15 (38.5) | 30 (76.9) | 60.67–88.87 | 120 vs. 160 | $\chi^2$ = 0.000<br>$P$ = 1.000 | | |
| 160 | 39 | 23 (59.0) | 30 (76.9) | 60.67–88.87 | P vs. Allo | $\chi^2$ = 52.478<br>$P$ < 0.001* | | |
| | | | | | Allo vs. 120 | $\chi^2$ = 0.652<br>$P$ < 0.420 | | |
| Allo | 38 | 11 (28.9) | 32 (84.2) | 68.75–93.98 | Allo vs. 160 | $\chi^2$ = 0.652<br>$P$ = 0.420 | — | |

#: Post hoc analysis (no statistical data)

* : $P$ < 0.05

P: Placebo group

120: Topiroxostat 120 mg group

160: Topiroxostat 160 mg group

Allo: Allopurinol group

Submitted to Clinical Rheumatology

Authors: Tatsuo Hosoya, Tomomitsu Sasaki, Tetsuo Ohashi

Corresponding author: Tomomitsu Sasaki

Development Department, Medical R&D Division, Fuji Yakuhin Co., Ltd.

tomomitsu@fujiyakuhin.co.jp
