# Supplementary material for: Clinical efficacy and safety of topiroxostat in Japanese hyperuricemic patients with or without gout: a randomized, double-blinded, controlled phase 2b study
Source: Clin Rheumatol. 2016 Nov 10;36(3):649–56. doi: 10.1007/s10067-016-3474-8 (PMC5323498; doi:10.1007/s10067-016-3474-8)
Supplement: Supplementary file 2 — (PDF 53 kb) [file 10067_2016_3474_MOESM2_ESM.pdf]

Table S2 Amount of change in serum urate level at the final visit from the level at baseline (FAS)

Group	n	Amount changes of serum urate level (μmol/L) mean ± SD	Statistics Tukey	
P	35	−22.4 ± 63.6	P vs 120	*P* < 0.001*
			P vs 160	*P* < 0.001*
120	39	−220.8 ± 64.7	120 vs 160	*P* = 0.559
160	39	−243.0 ± 82.1	Allo vs P	*P* < 0.001*
			Allo vs 120	*P* = 0.986
Allo	38	−226.7 ± 84.9	Allo vs 160	*P* = 0.773

*：*P* < 0.05

P: Placebo group

120: Topiroxostat 120 mg group

160: Topiroxostat 160 mg group

Allo: Allopurinol group

Clinical efficacy and safety of topiroxostat in Japanese hyperuricemic patients with or without gout: a randomized, double-blinded, controlled phase 2b study

Submitted to Clinical Rheumatology

Authors: Tatsuo Hosoya, Tomomitsu Sasaki, Tetsuo Ohashi

Corresponding author: Tomomitsu Sasaki

Development Department, Medical R&D Division, Fuji Yakuhin Co., Ltd.

tomomitsu@fujiyakuhin.co.jp
